# Supplementary material for: Three Rounds of Read Correction Significantly Improve Eukaryotic Protein Detection in ONT Reads
Source: Microorganisms. 2024 Jan 24;12(2):247. doi: 10.3390/microorganisms12020247 (PMC10893331; doi:10.3390/microorganisms12020247)
Supplement: Supplementary file 1 [file microorganisms-12-00247-s001.zip › microorganisms-2715757-supplementary.pdf]

microorganisms

MDPI

Table S1. The SRA numbers of sequencing reads (FASTQ), sequencing dates, base-call model used, and number f bases of all samples used in this study

Species	Sample number	SRA number	Sequencing Date	Caller Model Used	Number of Bases	Reference
C. albicans	Sample 1	SRR19900988	29-06-2022	Guppy (v6.0.1)	39.6M	[48]
	Sample 2	SRR19901033	29-06-2022	Guppy (v6.0.1)	74.8M	[48]
	Sample 3	SRR7874309	18-09-2018	Guppy (v6.0.1)	1.1G	[49]
	Sample 4	SRR21528967	19-12-2022	GridION X5 platform set to super accurate base calling	709.6M	[50]
	Sample 5	SRR17110952	08-12-2021	GridION	1.5G	N/A
	Sample 6	SRR17110953	08-12-2021	GridION	2.3G	N/A
C. gattii	Sample 1	SRR23862319	17-03-2023	N/A	77.9M	[51]
	Sample 2	SRR23862320	17-03-2023	N/A	244M	[51]
	Sample 3	SRR23862321	17-03-2023	N/A	221.5M	[51]
	Sample 4	SRR23862337	17-03-2023	N/A	308.3M	[51]
	Sample 5	SRR8435355	31-10-2019	Albacore (v2.3.1)	319.1M	[52]
	Sample 6	SRR8435356	31-10-2019	Albacore (v2.3.1)	604.9M	[52]
S. cerevisiae	Sample 1	ERR8609718	27-11-2022	Metrichor	1.4G	[53]
	Sample 2	ERR8562481	27-11-2022	Metrichor	1.6G	[53]
	Sample 3	ERR8562480	27-11-2022	Metrichor	1.3G	[53]
	Sample 4	ERR8562473	27-11-2022	Metrichor	593.7M	[53]
	Sample 5	ERR8562469	27-11-2022	Metrichor	811.9M	[53]
	Sample 6	ERR8562397	27-11-2022	Metrichor	857.5M	[53]
P. falciparum	Sample 1	ERR10160144	02-10-2022	MinIT (v18.09.1)	2.9G	[54]
	Sample 2	ERR10160143	02-10-2022	MinIT (v18.09.1)	1.2G	[54]
	Sample 3	ERR10160142	02-10-2022	MinIT (v18.09.1)	1.1G	[54]

	Sample 4	ERR10160141	02-10-2022	MinIT (v18.09.1)	902.7M	[54]
	Sample 5	ERR10502702	01-12-2022	Guppy (v6.0.7)	195.9M	[55]
	Sample 6	ERR10502698	01-12-2022	Guppy (v6.0.7)	135.1M	[55]

N/A: not applicable.

Table S2. NCBI references used for the four eukaryotic species, *C. albicans*, *C. gattii*, *S. cerevisiae,* and *P. falciparum.*

Species	Reference
C. albicansi	CP017623.1 *Candida albicans* SC5314
C. gattii	CP000286.1 *Cryptococcus gattii* WM276
S. cerevisiae	BK006935.2 TPA_inf: *Saccharomyces cerevisiae* S288C
P. falciparum	AL844501.2 *Plasmodium falciparum* 3D7

Table S3. Mapped reads percentage against the appropriate reference genome as detected by qualimap.

Species	Sample number	Coverage %
C. albicans	Sample 1	99.25
	Sample 2	88.57
	Sample 3	99.29
	Sample 4	99.59
	Sample 5	97.39
	Sample 6	99.23
C. gattii	Sample 1	98.53
	Sample 2	98.57
	Sample 3	91.7
	Sample 4	98.54
	Sample 5	93.93
	Sample 6	95.71
S. cerevisiae	Sample 1	99.45
	Sample 2	99.7
	Sample 3	97.53
	Sample 4	96.53
	Sample 5	96.53
	Sample 6	86.44
P. falciparum	Sample 1	99.77
	Sample 2	99.96
	Sample 3	99.94
	Sample 4	99.98
	Sample 5	100
	Sample 6	100
